# 2-Butyl-5-pentyl­benzene-1,3-diol

**DOI:** 10.1107/S1600536809018820

**Published:** 2009-05-23

**Authors:** Juangjun Jumpathong, Pascal Retailleau, Muna Ali Abdalla, Jamal Ouazzani, Saisamorn Lumyong

**Affiliations:** a Department of Biology, Faculty of Science, Chiang Mai University, Chiang Mai 50200, Thailand; b Institut de Chimie des Substances Naturelles - CNRS, 1 avenue de la Terrasse, 91198 Gif sur Yvette, France; c Department of Organic and Biomolecular Chemistry, Georg-August-Universität Göttingen, Tammannstrasse 2, D-37077 Göttingen, Germany

## Abstract

In the title compound, C_15_H_24_O_2_, a natural dialkyl­resorcinol commonly named stemphol, the mol­ecules are linked into *C*(6) and *C*_2_^2^(4) chains and *R*_4_^4^(16) rings by inter­molecular O—H⋯O hydrogen bonds, creating mol­ecular sheets parallel to the (010) plane. The alkyl chains are directed orthogonally away from these planes in almost complete extension.

## Related literature

For general background, synthesis, biological activity and related structures, see: Achenbach & Kohl (1979 ▶); Andersen & Frisvad (2004 ▶); Marumo *et al.* (1985 ▶); Solfrizzo *et al.* (1994 ▶); Stodola *et al.* (1973 ▶). For structural discussion, see: Etter (1990 ▶); Bernstein *et al.* (1995 ▶).
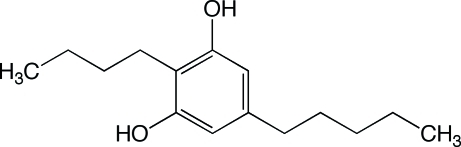


## Experimental

### 

#### Crystal data

C_15_H_24_O_2_
*M* *_r_* = 236.34
Monoclinic, 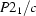

*a* = 4.654 (2) Å
*b* = 25.450 (5) Å
*c* = 12.790 (4) Å
β = 108.12 (1)°
*V* = 1439.8 (8) Å^3^
*Z* = 4
Mo *K*α radiation
μ = 0.07 mm^−1^
*T* = 293 K
0.50 × 0.10 × 0.08 mm

#### Data collection

Nonius KappaCCD diffractometer
Absorption correction: multi-scan (*SCALEPACK*; Otwinowski & Minor, 1997 ▶) *T*_min_ = 0.881, *T*_max_ = 0.994
15852 measured reflections
2631 independent reflections
1703 reflections with *I* > 2σ(*I*)
*R*_int_ = 0.029

#### Refinement

*R*[*F* ^2^ > 2σ(*F* ^2^)] = 0.048
*wR*(*F* ^2^) = 0.131
*S* = 1.04
2631 reflections
158 parameters
H-atom parameters constrained
Δρ_max_ = 0.18 e Å^−3^
Δρ_min_ = −0.16 e Å^−3^

### 

Data collection: *DENZO* (Otwinowski & Minor, 1997 ▶) and *COLLECT* (Nonius, 1999 ▶); cell refinement: *DENZO*; data reduction: *SCALEPACK* (Otwinowski & Minor, 1997 ▶); program(s) used to solve structure: *SIR97* (Altomare *et al.*, 1999 ▶); program(s) used to refine structure: *SHELXL97* (Sheldrick, 2008 ▶) and *CrystalBuilder* (Welter, 2006 ▶); molecular graphics: *PLATON* (Spek, 2009 ▶) and *Mercury* (Macrae *et al.*, 2006 ▶); software used to prepare material for publication: *SHELXL97* and *publCIF* (Westrip, 2009 ▶).

## Supplementary Material

Crystal structure: contains datablocks I, global. DOI: 10.1107/S1600536809018820/dn2455sup1.cif

Structure factors: contains datablocks I. DOI: 10.1107/S1600536809018820/dn2455Isup2.hkl

Additional supplementary materials:  crystallographic information; 3D view; checkCIF report

## Figures and Tables

**Table 1:** Hydrogen-bond geometry (Å, °)

*D*—H⋯*A*	*D*—H	H⋯*A*	*D*⋯*A*	*D*—H⋯*A*
O1—H1⋯O2^i^	0.82	1.96	2.767 (2)	167
O2—H2⋯O1^ii^	0.82	1.95	2.750 (2)	165

Symmetry codes: (i) 
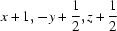
; (ii) 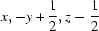.

**Fig. 1.**
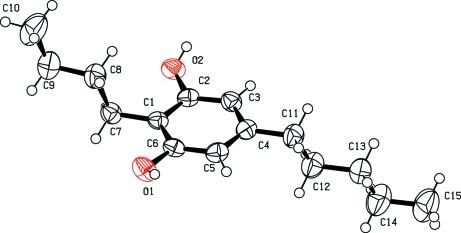
Molecular view of the compound with the atom labelling scheme. Displacement ellipsoids are drawn at the 50% probability level. H atoms are represented as small spheres of arbitrary radii.

**Fig. 2.**
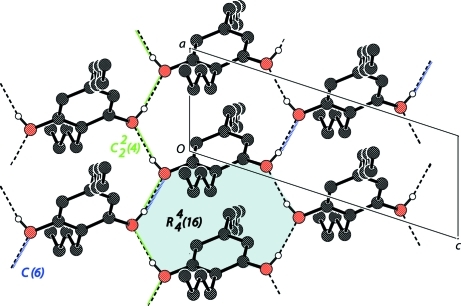
The crystal packing of the title molecule, viewed down the b axis, showing the molecules connected by O—H···O hydrogen bonds in dotted lines. H atoms not involved in hydrogen bondings have been omitted for clarity.

**Fig. 3.**
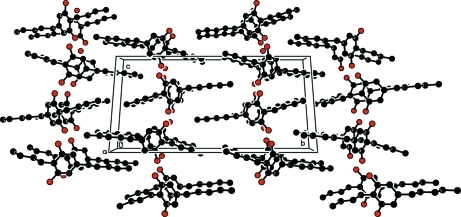
The crystal packing of the title molecule, viewed down the a axis, showing the segregration between alkyl chains and resorcinol moieties; H atoms are omitted for clarity.

## supplementary crystallographic information

### Comment

Stemphol was first isolated from *Stemphylium majusculum* (Stodola *et al.*, 1973) and its related compounds were reported with antimicrobial activities against fungus (*Mucor hiemalis*), yeast (*Schizosaccharomyces pombe*) and Gram positive bacteria (*Bacillus subtilis* and *Staphylococcus aureus*) (Achenbach & Kohl, 1979). Later on, stemphol was isolated from *Pleospora herbarum* and described as self-inhibitor (Marumo *et al.*, 1985). Five of eleven isolates of *Stemphylium botryosum* Wallr. from oilseed rape produced the phytotoxin, stemphol when cultured on rice (Solfrizzo *et al.*, 1994). *Stemphylium cf. lycopersici* was postharvest spoiler of fresh tomatoes and was first detected of stemphols in naturally contaminated tomatoes (Andersen & Frisvad, 2004). Screening investigation for polyketide and novel substance from new endophytic fungus led to the finding of stemphol from *Gaeumannomyces amomi* BCC4066 isolated from healthy pseudostem of *Alpinia malaccensis*, which was collected in the Suthep- Pui National Park, Chiang Mai, in the northern part of Thailand. This molecule has been reported for the first time in endophytic fungus and its X-ray structure (Fig. 1) is presented herein.

The two hydroxyl groups of the stemphol molecules lead to the formation of chains and rings through O—H···O hydrogen bonds (Table 1) with graph set motifs C(6), C_2_^2^(4) and *R*_4_^4^(16) (Etter, 1990; Bernstein *et al.*, 1995) all contained within the glide planes (Fig. 2). Both butyl and pentyl chains, directed in opposite directions and running up and down with respect to the resorcinol mean plane, adopt essentially fully extended conformations, but with 12.7 (2)° of deviation between their mean planes defined by C1–C7/C10 and C4–C12/C15 respectively (Fig. 3). The dihedral angles they form with respect to the (010) plane are 84.3 (2)° and 73.5 (2)° respectively.

### Experimental

*Gaeumannomyces amomi* BCC4066 was cultivated in 20 l of liquid culture and incubated for 21 d at room temperature (20 °C). Liquid culture was filtrated and extracted separately. The residue (5.0 g) obtained after evaporation of solvent was subjected to column chromatography over sephadex to afford 30 mg of Stemphol. Colourless needle-shaped crystals were obtained by re-crystallization from 20% EtOAc in Heptane (m.p. 91 °C).

### Refinement

All H atoms were located in difference maps but were treated as riding on their parent atoms, with O—H = 0.82 Å, and C—H distances in the range 0.93–0.97 Å and with *U*_iso_(H) = 1.2*U*_eq_(carrier) [1.5 for methyl H atoms].

### Crystal data

**Table:**

C_15_H_24_O_2_	*F*(000) = 520
*M**_r_* = 236.34	*D*_x_ = 1.090 Mg m^−^^3^
Monoclinic, *P*2_1_/*c*	Melting point: 364 K
Hall symbol: -P 2ybc	Mo *K*α radiation, λ = 0.71069 Å
*a* = 4.654 (2) Å	Cell parameters from 2700 reflections
*b* = 25.450 (5) Å	θ = 0.4–25.4°
*c* = 12.790 (4) Å	µ = 0.07 mm^−^^1^
β = 108.12 (1)°	*T* = 293 K
*V* = 1439.8 (8) Å^3^	Needle, colourless
*Z* = 4	0.50 × 0.10 × 0.08 mm

### Data collection

**Table:**

Nonius KappaCCD diffractometer	2631 independent reflections
Radiation source: fine-focus sealed tube	1703 reflections with *I* > 2σ(*I*)
graphite	*R*_int_ = 0.029
φ and ω scans	θ_max_ = 25.3°, θ_min_ = 2.3°
Absorption correction: multi-scan (*SCALEPACK*; Otwinowski & Minor, 1997)	*h* = −5→5
*T*_min_ = 0.881, *T*_max_ = 0.994	*k* = −30→30
15852 measured reflections	*l* = −15→15

### Refinement

**Table:**

Refinement on *F*^2^	Primary atom site location: structure-invariant direct methods
Least-squares matrix: full	Secondary atom site location: difference Fourier map
*R*[*F*^2^ > 2σ(*F*^2^)] = 0.048	Hydrogen site location: difference Fourier map
*wR*(*F*^2^) = 0.131	H-atom parameters constrained
*S* = 1.04	*w* = 1/[σ^2^(*F*_o_^2^) + (0.0625*P*)^2^ + 0.1805*P*] where *P* = (*F*_o_^2^ + 2*F*_c_^2^)/3
2631 reflections	(Δ/σ)_max_ < 0.001
158 parameters	Δρ_max_ = 0.18 e Å^−^^3^
0 restraints	Δρ_min_ = −0.15 e Å^−^^3^

### Special details

**Table:**

Geometry. All e.s.d.'s (except the e.s.d. in the dihedral angle between two l.s. planes) are estimated using the full covariance matrix. The cell e.s.d.'s are taken into account individually in the estimation of e.s.d.'s in distances, angles and torsion angles; correlations between e.s.d.'s in cell parameters are only used when they are defined by crystal symmetry. An approximate (isotropic) treatment of cell e.s.d.'s is used for estimating e.s.d.'s involving l.s. planes.
Refinement. Refinement of *F*^2^ against ALL reflections. The weighted *R*-factor *wR* and goodness of fit *S* are based on *F*^2^, conventional *R*-factors *R* are based on *F*, with *F* set to zero for negative *F*^2^. The threshold expression of *F*^2^ > σ(*F*^2^) is used only for calculating *R*-factors(gt) *etc*. and is not relevant to the choice of reflections for refinement. *R*-factors based on *F*^2^ are statistically about twice as large as those based on *F*, and *R*-factors based on ALL data will be even larger.

### Fractional atomic coordinates and isotropic or equivalent isotropic displacement parameters (Å^2^)

**Table:**

	*x*	*y*	*z*	*U*_iso_*/*U*_eq_	
O1	0.0951 (3)	0.27129 (5)	0.28490 (10)	0.0555 (4)	
H1	0.2648	0.2719	0.3299	0.083*	
O2	−0.2986 (2)	0.22721 (5)	−0.09324 (9)	0.0517 (4)	
H2	−0.2074	0.2253	−0.1386	0.077*	
C1	−0.1145 (3)	0.24879 (6)	0.09661 (13)	0.0358 (4)	
C2	−0.1012 (3)	0.21631 (7)	0.01107 (13)	0.0383 (4)	
C3	0.0994 (4)	0.17495 (7)	0.02531 (14)	0.0428 (4)	
H3	0.1000	0.1542	−0.0345	0.051*	
C4	0.3006 (3)	0.16426 (7)	0.12888 (14)	0.0414 (4)	
C5	0.2925 (3)	0.19620 (7)	0.21530 (14)	0.0432 (5)	
H5	0.4233	0.1896	0.2855	0.052*	
C6	0.0935 (3)	0.23774 (7)	0.19898 (13)	0.0394 (4)	
C7	−0.3211 (4)	0.29564 (7)	0.07705 (15)	0.0454 (5)	
H7A	−0.3674	0.3038	0.1441	0.054*	
H7B	−0.5092	0.2871	0.0207	0.054*	
C8	−0.1801 (4)	0.34346 (7)	0.04135 (17)	0.0557 (5)	
H8A	0.0020	0.3528	0.0999	0.067*	
H8B	−0.1210	0.3341	−0.0226	0.067*	
C9	−0.3832 (5)	0.39095 (8)	0.0136 (2)	0.0756 (7)	
H9A	−0.5625	0.3822	−0.0468	0.091*	
H9B	−0.4472	0.3999	0.0766	0.091*	
C10	−0.2332 (6)	0.43810 (9)	−0.0182 (3)	0.1069 (10)	
H10A	−0.0721	0.4500	0.0445	0.160*	
H10B	−0.3790	0.4657	−0.0430	0.160*	
H10C	−0.1529	0.4286	−0.0762	0.160*	
C11	0.5085 (4)	0.11754 (7)	0.14655 (16)	0.0523 (5)	
H11A	0.6758	0.1228	0.2134	0.063*	
H11B	0.5910	0.1151	0.0858	0.063*	
C12	0.3515 (4)	0.06650 (7)	0.15543 (18)	0.0604 (6)	
H12A	0.1767	0.0628	0.0905	0.073*	
H12B	0.2783	0.0687	0.2185	0.073*	
C13	0.5435 (4)	0.01760 (7)	0.16697 (17)	0.0594 (5)	
H13A	0.6171	0.0153	0.1040	0.071*	
H13B	0.7178	0.0211	0.2322	0.071*	
C14	0.3833 (5)	−0.03251 (8)	0.1752 (2)	0.0848 (8)	
H14A	0.3137	−0.0304	0.2391	0.102*	
H14B	0.2060	−0.0354	0.1109	0.102*	
C15	0.5672 (6)	−0.08146 (9)	0.1840 (2)	0.0970 (9)	
H15A	0.6319	−0.0848	0.1200	0.145*	
H15B	0.4468	−0.1114	0.1892	0.145*	
H15C	0.7409	−0.0796	0.2485	0.145*	

### Atomic displacement parameters (Å^2^)

**Table:**

	*U*^11^	*U*^22^	*U*^33^	*U*^12^	*U*^13^	*U*^23^
O1	0.0453 (7)	0.0768 (9)	0.0399 (8)	0.0016 (7)	0.0069 (6)	−0.0146 (7)
O2	0.0446 (7)	0.0744 (9)	0.0314 (7)	0.0080 (6)	0.0052 (5)	0.0047 (7)
C1	0.0292 (8)	0.0443 (10)	0.0339 (9)	−0.0028 (7)	0.0099 (7)	0.0024 (8)
C2	0.0316 (8)	0.0488 (10)	0.0311 (9)	−0.0041 (8)	0.0046 (7)	0.0039 (8)
C3	0.0432 (9)	0.0484 (11)	0.0367 (10)	0.0004 (9)	0.0120 (8)	−0.0031 (8)
C4	0.0345 (8)	0.0442 (10)	0.0442 (11)	−0.0015 (8)	0.0104 (8)	0.0053 (8)
C5	0.0333 (9)	0.0552 (11)	0.0350 (10)	−0.0007 (8)	0.0018 (7)	0.0063 (9)
C6	0.0342 (9)	0.0511 (11)	0.0325 (10)	−0.0050 (8)	0.0101 (7)	−0.0029 (8)
C7	0.0369 (9)	0.0538 (11)	0.0450 (11)	0.0028 (8)	0.0119 (8)	−0.0009 (9)
C8	0.0491 (10)	0.0527 (12)	0.0640 (13)	0.0043 (9)	0.0156 (9)	0.0016 (10)
C9	0.0815 (15)	0.0596 (14)	0.0889 (17)	0.0159 (12)	0.0310 (13)	0.0062 (13)
C10	0.131 (2)	0.0567 (16)	0.138 (3)	0.0108 (15)	0.050 (2)	0.0224 (16)
C11	0.0440 (10)	0.0511 (11)	0.0592 (12)	0.0069 (9)	0.0124 (8)	0.0052 (10)
C12	0.0517 (11)	0.0524 (12)	0.0753 (14)	0.0037 (9)	0.0168 (10)	0.0068 (11)
C13	0.0604 (12)	0.0521 (12)	0.0638 (14)	0.0075 (10)	0.0167 (10)	0.0006 (10)
C14	0.0828 (16)	0.0543 (14)	0.113 (2)	0.0030 (12)	0.0242 (15)	0.0058 (13)
C15	0.118 (2)	0.0560 (15)	0.113 (2)	0.0078 (14)	0.0293 (17)	−0.0002 (14)

### Geometric parameters (Å, °)

**Table:**

O1—C6	1.3899 (19)	C9—H9A	0.9700
O1—H1	0.8200	C9—H9B	0.9700
O2—C2	1.3924 (18)	C10—H10A	0.9600
O2—H2	0.8200	C10—H10B	0.9600
C1—C2	1.388 (2)	C10—H10C	0.9600
C1—C6	1.394 (2)	C11—C12	1.512 (3)
C1—C7	1.503 (2)	C11—H11A	0.9700
C2—C3	1.381 (2)	C11—H11B	0.9700
C3—C4	1.391 (2)	C12—C13	1.512 (3)
C3—H3	0.9300	C12—H12A	0.9700
C4—C5	1.382 (2)	C12—H12B	0.9700
C4—C11	1.505 (2)	C13—C14	1.497 (3)
C5—C6	1.378 (2)	C13—H13A	0.9700
C5—H5	0.9300	C13—H13B	0.9700
C7—C8	1.519 (3)	C14—C15	1.496 (3)
C7—H7A	0.9700	C14—H14A	0.9700
C7—H7B	0.9700	C14—H14B	0.9700
C8—C9	1.507 (3)	C15—H15A	0.9600
C8—H8A	0.9700	C15—H15B	0.9600
C8—H8B	0.9700	C15—H15C	0.9600
C9—C10	1.506 (3)		
			
C6—O1—H1	109.5	H9A—C9—H9B	107.8
C2—O2—H2	109.5	C9—C10—H10A	109.5
C2—C1—C6	115.59 (15)	C9—C10—H10B	109.5
C2—C1—C7	121.63 (14)	H10A—C10—H10B	109.5
C6—C1—C7	122.55 (15)	C9—C10—H10C	109.5
C3—C2—C1	122.97 (15)	H10A—C10—H10C	109.5
C3—C2—O2	119.73 (15)	H10B—C10—H10C	109.5
C1—C2—O2	117.29 (14)	C4—C11—C12	112.77 (14)
C2—C3—C4	120.13 (16)	C4—C11—H11A	109.0
C2—C3—H3	119.9	C12—C11—H11A	109.0
C4—C3—H3	119.9	C4—C11—H11B	109.0
C5—C4—C3	117.98 (15)	C12—C11—H11B	109.0
C5—C4—C11	121.38 (16)	H11A—C11—H11B	107.8
C3—C4—C11	120.57 (17)	C11—C12—C13	115.46 (16)
C6—C5—C4	120.95 (15)	C11—C12—H12A	108.4
C6—C5—H5	119.5	C13—C12—H12A	108.4
C4—C5—H5	119.5	C11—C12—H12B	108.4
C5—C6—O1	120.90 (14)	C13—C12—H12B	108.4
C5—C6—C1	122.35 (16)	H12A—C12—H12B	107.5
O1—C6—C1	116.74 (14)	C14—C13—C12	114.58 (17)
C1—C7—C8	111.85 (14)	C14—C13—H13A	108.6
C1—C7—H7A	109.2	C12—C13—H13A	108.6
C8—C7—H7A	109.2	C14—C13—H13B	108.6
C1—C7—H7B	109.2	C12—C13—H13B	108.6
C8—C7—H7B	109.2	H13A—C13—H13B	107.6
H7A—C7—H7B	107.9	C15—C14—C13	115.4 (2)
C9—C8—C7	114.57 (16)	C15—C14—H14A	108.4
C9—C8—H8A	108.6	C13—C14—H14A	108.4
C7—C8—H8A	108.6	C15—C14—H14B	108.4
C9—C8—H8B	108.6	C13—C14—H14B	108.4
C7—C8—H8B	108.6	H14A—C14—H14B	107.5
H8A—C8—H8B	107.6	C14—C15—H15A	109.5
C10—C9—C8	113.16 (18)	C14—C15—H15B	109.5
C10—C9—H9A	108.9	H15A—C15—H15B	109.5
C8—C9—H9A	108.9	C14—C15—H15C	109.5
C10—C9—H9B	108.9	H15A—C15—H15C	109.5
C8—C9—H9B	108.9	H15B—C15—H15C	109.5
			
C6—C1—C2—C3	1.2 (2)	C7—C1—C6—C5	−176.77 (15)
C7—C1—C2—C3	175.85 (15)	C2—C1—C6—O1	176.89 (14)
C6—C1—C2—O2	−177.88 (13)	C7—C1—C6—O1	2.3 (2)
C7—C1—C2—O2	−3.2 (2)	C2—C1—C7—C8	−83.9 (2)
C1—C2—C3—C4	−0.1 (2)	C6—C1—C7—C8	90.4 (2)
O2—C2—C3—C4	179.01 (14)	C1—C7—C8—C9	176.41 (17)
C2—C3—C4—C5	−0.2 (2)	C7—C8—C9—C10	178.26 (19)
C2—C3—C4—C11	176.65 (15)	C5—C4—C11—C12	98.0 (2)
C3—C4—C5—C6	−0.8 (2)	C3—C4—C11—C12	−78.7 (2)
C11—C4—C5—C6	−177.58 (15)	C4—C11—C12—C13	176.76 (17)
C4—C5—C6—O1	−177.00 (15)	C11—C12—C13—C14	−179.8 (2)
C4—C5—C6—C1	2.0 (2)	C12—C13—C14—C15	178.6 (2)
C2—C1—C6—C5	−2.2 (2)		

### Hydrogen-bond geometry (Å, °)

**Table:**

*D*—H···*A*	*D*—H	H···*A*	*D*···*A*	*D*—H···*A*
O1—H1···O2^i^	0.82	1.96	2.767 (2)	167
O2—H2···O1^ii^	0.82	1.95	2.750 (2)	165

Symmetry codes: (i) *x*+1, −*y*+1/2, *z*+1/2; (ii) *x*, −*y*+1/2, *z*−1/2.
